# Human Motor System‐Based Biohybrid Robot‐On‐a‐Chip for Drug Evaluation of Neurodegenerative Disease

**DOI:** 10.1002/advs.202305371

**Published:** 2023-11-30

**Authors:** Minkyu Shin, Taehyeong Ha, Joungpyo Lim, Joohyun An, Geunyoung Beak, Jin‐Ha Choi, Ambrose Ashwin Melvin, Jinho Yoon, Jeong‐Woo Choi

**Affiliations:** ^1^ Department of Chemical & Biomolecular Engineering Sogang University 35 Baekbeom‐ro, Mapo‐gu Seoul 04107 Republic of Korea; ^2^ School of Chemical Engineering Jeonbuk National University 567 Baekje‐daero, Deokjin‐gu Jeonju‐si Jeollabuk‐do 54896 Republic of Korea; ^3^ Department of Biomedical‐Chemical Engineering The Catholic University of Korea 43 Jibong‐ro, Wonmi‐gu Bucheon‐si Gyeonggi‐do 14662 Republic of Korea; ^4^ Department of Biotechnology The Catholic University of Korea 43 Jibong‐ro, Wonmi‐gu Bucheon‐si Gyeonggi‐do 14662 Republic ofKorea

**Keywords:** biohybrid robot‐on‐a‐chip, brain organoid, human motor system, motor neuron spheroids, muscle bundle, neurodegenerative disease

## Abstract

Biohybrid robots have been developed for biomedical applications and industrial robotics. However, the biohybrid robots have limitations to be applied in neurodegenerative disease research due to the absence of a central nervous system. In addition, the organoids‐on‐a‐chip has not yet been able to replicate the physiological function of muscle movement in the human motor system, which is essential for evaluating the accuracy of the drugs used for treating neurodegenerative diseases. Here, a human motor system‐based biohybrid robot‐on‐a‐chip composed of a brain organoid, multi‐motor neuron spheroids, and muscle bundle on solid substrateis proposed to evaluate the drug effect on neurodegenerative diseases for the first time. The electrophysiological signals from the cerebral organoid induced the muscle bundle movement through motor neuron spheroids. To evaluate the drug effect on Parkinson's disease (PD), a patient‐derived midbrain organoid is generated and incorporated into a biohybrid robot‐on‐a‐chip. The drug effect on PD is successfully evaluated by measuring muscle bundle movement. The muscle bundle movement of PD patient‐derived midbrain organoid‐based biohybrid robot‐on‐a‐chip is increased from 4.5 ± 0.99 µm to 18.67 ± 2.25 µm in response to levodopa. The proposed human motor system‐based biohybrid robot‐on‐a‐chip can serve as a standard biohybrid robot model for drug evaluation.

## Graphical abstract

Human motor system‐based biohybrid robot‐on‐a‐chip composed of a brain organoid, multi‐motor neuron spheroids, and muscle bundle on solid substrate is proposed to evaluate the drug effect on neurodegenerative diseases. The electrophysiological signals from the cerebral organoid induce the muscle bundle movement through the connected motor neuron spheroids. The drug effect on Parkinson's disease is evaluated by measuring muscle bundle movement.

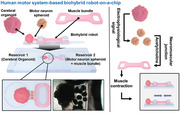

## 1 Introduction

Biohybrid robots, which comprise biological components and soft materials, have recently attracted huge attention in biomedical applications and industrial robotics owing to their environmental adaptability, similarity to living organisms, and capacity for efficient energy conversion.^[^ ^1^, ^2^ ^]^ Besides, unlike polymer‐based soft robots, biohybrid robots are composed of biological materials and, thus, have high biocompatibility.^[^ ^3^ ^]^ In addition, biohybrid robots have great potential in biomedical applications such as disease model development and drug evaluation because they can mimic in vivo microstructures and exhibit human‐like tissue or organ functions.^[^ ^4^ ^]^ Despite its potential for biomedical applications, there are still few reports of drug evaluation using the movement of the biohybrid robot practically.^[^ ^5^, ^6^ ^]^ However, the biohybrid robots developed so far are still actuated by spontaneous movement or electrical/optical muscle stimulation.^[^ ^7^, ^8^, ^9^ ^]^ Specifically, since these biohybrid robots do not have brain‐based central nervous system (CNS), they have difficulty mimicking a human motor system, in which muscle movement is controlled by the brain. In the operation of an actual human motor system, electrophysiological signals generated from the CNS are transmitted to skeletal muscles through connected motor nerves to induce muscle movement.^[^ ^10^, ^11^ ^]^ Moreover, the current biohybrid robots, being composed of muscle cells/motor neurons, can only be used for developing muscle or neuromuscular disease models.^[^ ^12^, ^13^ ^]^ To address this limitation, we develop the biohybrid robot composed of brain organoid, motor neuron spheroid, and muscle bundle. In addition, we propose a human motor system‐based biohybrid robot‐on‐a‐chip composed of a brain organoid, multi‐motor neuron spheroids, and muscle bundle on the solid substrate similar to human‐on‐a‐chip or organoids‐on‐a‐chip (OoC) for accurate drug evaluation on neurodegenerative diseases.

Recently, as a promising way to evaluate the drug effect to diseases, the OoC models were designed to simulate human organs and develop accurate drug evaluation models.^[^ ^14^, ^15^, ^16^ ^]^ OoC is a biomimetic system that uses microfabrication techniques to develop miniature 3D models of human organs and organ systems on a microchip.^[^ ^17^ ^]^ These OoCs can be used to simulate human organs, providing more efficient alternatives to traditional animal testing for drug development, toxicology, and biological medicine research.^[^ ^15^, ^18^ ^]^ In addition, it mimics the microenvironment of human organs, enabling researchers to study pathogenesis and the effects of drugs on different organs. So far, several OoC models have been developed for mimicking the organ system, including the structure and functions of the liver, gut, and lung.^[^ ^19^, ^20^, ^21^ ^]^ However, OoC has not yet been able to replicate the physiological function of muscle movement in the human motor system, which is essential for evaluating the accuracy of the drugs used for treating neurodegenerative diseases, which occur in the CNS and adversely affect muscle movement. Parkinson's disease (PD) is a neurodegenerative disease that is caused by the degeneration of dopaminergic neurons in the brain.^[^ ^22^ ^]^ When dopaminergic neurons are damaged, the amount of dopamine available in the brain decreases, leading to a disruption in muscle movement.^[^ ^23^ ^]^ From this point of view, the development of the human motor system‐based biohybrid robot‐on‐a‐chip with response to chemical stimulation by introducing the CNS such as the brain into a biohybrid robot is a promising strategy for evaluating the effects of different drugs on neurodegenerative diseases.

**Figure 1:**
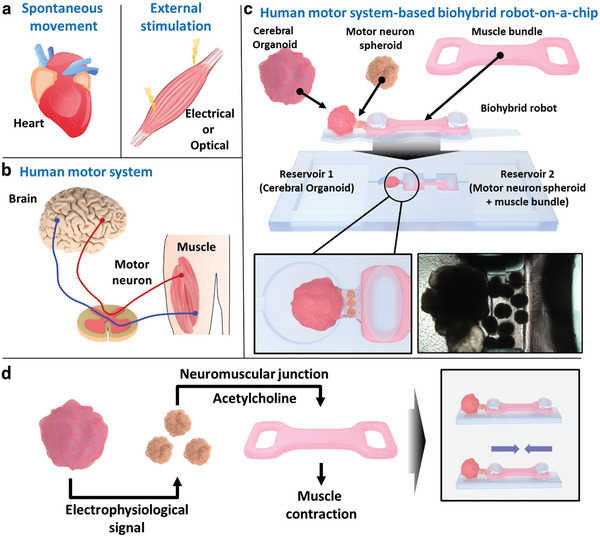
Schematic illustration of the human motor system‐based biohybrid robot‐on‐a‐chip. a) Schematic images of spontaneous movement and muscle movement induced by external stimulation. b) Schematic image of the human motor system. c) Schematic representation of the human motor system‐based biohybrid robot‐on‐a‐chip composed of the cerebral organoid, motor neuron spheroid (MNS), and muscle bundle on the solid substrate. d) Schematic diagram representing muscle bundle movement through the connected cerebral organoid and multi‐MNSs.

Here, a human motor system‐based biohybrid robot‐on‐a‐chip composed of brain organoid, motor neuron spheroid (MNS), and muscle bundle on solid substrate was developed for the first time to evaluate the effects of drugs on neurodegenerative disease like PD. As shown in **Figure** **1**, the human induced pluripotent stem cell (iPSC)‐derived cerebral organoid, human neural stem cell (NSC)‐derived MNSs, and skeletal muscle bundle were connected and prepared on the 3D‐printed polymer substrate to develop a biohybrid robot‐on‐a‐chip. Furthermore, the MNSs in this biohybrid robot‐on‐a‐chip were mixed with the human umbilical vein endothelial cells (HUVECs) and hyaluronic acid (HA)‐modified Au‐Ni‐Au nanorods (HA@ANA NRs) to improve the connection between the cerebral organoid and muscle bundle. During the connection between cerebral organoid and muscle bundle, the HUVECs positively affected neuron cells, enhancing neuronal proliferation and neurogenesis. In particular, the vascular network supported neuron outgrowth and neural network formation by efficiently supplying oxygen and nutrients. Furthermore, HA@ANA NRs promoted the magnetic field‐assisted alignment of the growing neurites of the MNS via their magnetic component (Nickel). The fabricated human motor system‐based biohybrid robot‐on‐a‐chip indicated muscle bundle movement through electrophysiological signals produced by neurotransmitters in the cerebral organoid, which were successfully transmitted through MNSs to the muscle bundle. In addition, PD patient‐derived midbrain organoid (PD‐midbrain organoid) was generated to develop a PD‐midbrain organoid‐based biohybrid robot‐on‐a‐chip to evaluate the effects of drugs on neurodegenerative diseases.

## 2 Results and Discussion

### 2.1 Generation and Characterization of the Cerebral Organoid, MNS, and Muscle Bundle

**Figure 2:**
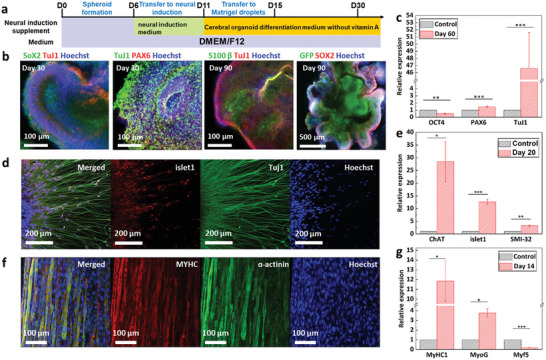
Characterization of cerebral organoid, motor neuron spheroid, and muscle bundle. a) Graphical protocol for generating cerebral organoids. b) Immunostaining images of the cerebral organoid. c) Normalized gene expression levels of OCT4, PAX6, and TuJ1 in the cerebral organoid. *P < 0.05, **P < 0.01, and ***P < 0.001. Error bars correspond to the standard deviation (SD) from three measurements. d) Immunostaining images of the differentiated motor neuron on day 28. Scale bar: 200 µm. e) Normalized expression levels of ChAT, islet1, and SMI‐32 in MNSs. *P < 0.05, **P < 0.01, and ***P < 0.001. Error bars correspond to the SD from five measurements. f) Immunostaining image of muscle bundle for verifying myogenic differentiation on day 14. g) Normalized gene expression levels of MyHC1, MyoG, and Myf5 in the muscle bundle. *P < 0.05, **P < 0.01, and ***P < 0.001. Error bars correspond to the SD from four measurements.

A human cerebral organoid for the biohybrid robot was generated from human iPSCs, as described in Lancaster's protocol (**Figure** **2**a).^[^ ^24^ ^]^ As shown in Figure S1a (Supporting Information), the cerebral organoid was generated using the embryoid body (EB) and neuroepithelial tissue. The outer bright and inner dark tissues of the EBs were usually more clearly distinguishable at 8–10 d after EB generation. After the outer bright part of the neuroectodermal tissue and the well‐organized neuroectoderm formed smoothly around the surface, the neuroectodermal tissue was embedded in a Matrigel droplet to provide a scaffold for growth into neuroepithelial tissue. Next, the cerebral organoid maturated more than 3 mm in diameter with multiple neuroepithelial tissues (Figure S1b, Supporting Information). To confirm the generation of a cerebral organoid, immunostaining analysis was performed using SOX2, PAX6, TuJ1, and S100β, which are biomarkers for progenitor cells, neurons, and astrocytes in a generated cerebral organoid (Figure 2b). Immunostaining of the radial glial markers SOX2 and PAX6 on day 30 indicated that the cerebral organoid contained a higher number of radial glial cells along the ventricular zone (VZ). The neuronal marker TuJ1 was also found to form around the VZ, and the mature glial cell marker S100β indicated the neurons comprising the cerebral organoid. In addition, to investigate the expression levels of the biomarkers (OCT4, PAX6, and TuJ1), quantitative polymerase chain reaction (qPCR) was performed using the cerebral organoid on days 0, 30, and, 60 (Figure 2c). The qPCR analysis indicated that the expression of these three markers in the cerebral organoid was 1.212, 1.388, and 1.584 times higher than that in the undifferentiated human iPSCs (Control). These results verified that the cerebral organoid was successfully generated using human iPSCs. Furthermore, the electrophysiological activity of the cerebral organoid was confirmed using the multielectrode array (MEA) system on days 30 and 90 (Figure S2, Supporting Information). The number of spikes in the cerebral organoid increased from 21.0 spikes min^−1^ on day 30 to 68.16 spikes min^−1^ on day 90, and bursts were generated from the cerebral organoid only on day 90.

To generate the MNS, human NSCs (6.0 × 10^4^ cells per well) were seeded on a round‐bottom 96‐well plate to spontaneously form human NSC‐based spheroids and cell aggregation was observed in the well plate. The formed spheroids were guided to differentiate and mature into MNSs for 28 , as mentioned in a previous study.^[^ ^25^ ^]^ To observe the neurons in the differentiated MNS, immunostaining of a typical motor neuron marker (islet1) and a neuronal marker (TuJ1) of the differentiated MNS was conducted (Figure 2d). In addition, to confirm MNS differentiation, the expression levels of motor neuron differentiation markers islet1, SMI‐32, and ChAT were measured using qPCR (Figure 2e). The gene expression levels of SMI‐32, islet1, and ChAT were 3.29, 12.594, and 28.48 times higher, respectively, in the differentiated spheroid than in the control spheroids (Differentiation day 1).

The differentiation of the muscle bundle was confirmed by immunostaining sarcomeric α‐actinin (Muscle differentiation marker) and myosin heavy chain (MyHC). The differentiated muscle bundle showed a high expression of both sarcomeric α‐actinin and MyHC (Figure 2f). In addition, qPCR was performed using the muscle bundle to investigate the expression levels of myogenic gene markers MyHC1, myogenin (MyoG), and myogenic factor 5 (Myf5). The gene expression levels of MyCH1 and MyoG in differentiated muscle bundle were upregulated by 11.84 and 3.75 times, respectively, whereas that of Myf5 was downregulated by approximately 0.19 times, compared to those in the undifferentiated muscle cells (Figure 2g). These results verified that the cerebral organoid, MNS, and muscle bundle were successfully generated for use in the development of a human motor system‐based biohybrid robot‐on‐a‐chip.

### 2.2 Confirmation of the Improvement in Cell Elongation by HA@ANA NRs and HUVECs

**Figure 3:**
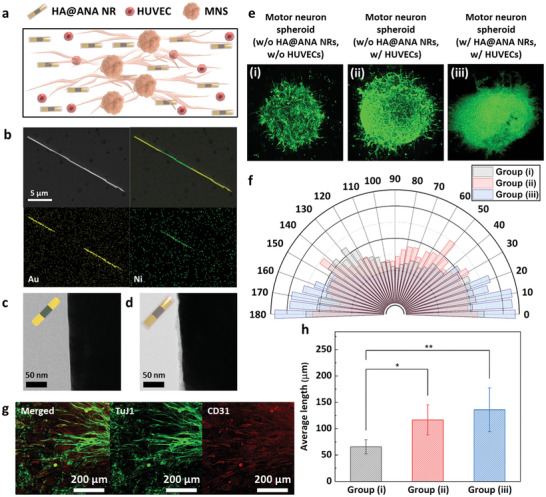
Effect of HA@ANA NRs and HUVECs on the motor neuron. a) Schematic image of multi‐MNSs with HA@ANA NRs and HUVECs. b) SEM image and EDS mapping image of ANA NRs. TEM images of c) ANA NRs and d) HA@ANA NRs. e) Fluorescent images and f) quarter‐circle graphs showing the distribution of the directionality of the neurites of the motor neuron of three groups: (i) MNS without HA@ANA NRs and HUVECs, (ii) MNS without HA@ANA NRs, and (iii) MNS with HA@ANA NRs and HUVECs. g) Images of vascular network formation obtained after immunostaining of TuJ1 (Green) and CD31 (Red). h) Quantification of the average length of neurite. *P < 0.05 and **P < 0.01. Error bars correspond to the SD from five measurements.

To connect the cerebral organoid, multi‐MNSs, and muscle bundle to develop a biohybrid robot‐on‐a‐chip, first, the HUVECs and HA@ANA NRs were introduced on extracellular matrix (ECM) hydrogel (**Figure** **3**a). The ANA NRs were synthesized through electrochemical deposition of Au and Ni inside the 0.2‐µm sized pores of the anodized aluminum oxide (AAO) template (Figure S3a, Supporting Information). The field emission scanning electron microscopy (FE‐SEM) image revealed that the synthesized ANA NR had Ni and Au components (Figure 3b and Figure S3b–d, Supporting Information). In addition, the biocompatibility of ANA NRS was enhanced by using thiol‐modified HA to functionalize the surface of ANA NRs with gold–thiol interactions to produce the HA@ANA NRs. HA plays an important role in 3D cell culture and proliferation owing to its high capacity for water retention and providing structural support. The synthesis of HA@ ANA NRs was confirmed using a transmission electron microscope (TEM) (Figure 3c,d). Compared to ANA NRs, HA@ANA NRs had a 10 nm thin layer of HA immobilized on the ANA surface. We also found that HA is immobilized on the Ni surface as well as the Au surface of the ANA NR, and that the C component of HA indicated over the entire area of the ANA NR (Figure S4a, Supporting Information). Furthermore, the zeta potential reduced from −2.11 ± 1.39 mV in ANA NRs to −56.31 ± 9.54 mV in HA@ANA NRs owing to the negative charge of HA (Figure S4b, Supporting Information). The synthesized HA@ANA NRs were easily aligned within the Matrigel due to the Ni component (Figure S5a,b, Supporting Information). In addition, as shown in Figure S5c (Supporting Information), the alignment value in 0–10° was indicated 2.17 times higher values compared to before the alignment.

Next, the HA@ANA NRs were mixed with HUVECs and combined with a MNS to induce the proper alignment and growth of the MNS. To determine the effect of HA@ANA NRs and HUVECs on neurite formation in the MNS, three groups were separately cultured on laminin‐coated 35 mm dishes: i) MNS without HA@ANA NRs and HUVECs, ii) MNS without HA@ANA NRs, and iii) MNS with HA@ANA NRs and HUVECs. Subsequently, the formation and alignment of neurites in each group were confirmed on day 5 using fluorescence microscopy (Figure 3e). As shown in Figure 3f, group (iii) showed 1.34 times higher values in 0–10° compared to group (i) because of magnet‐assisted alignment of HA@ANA NRs using their Ni component. In addition, for groups (ii) and (iii), a 1:1 mixture of HUVEC growth medium and MNS differentiation medium was used at the beginning of the co‐culture for vascular network formation. The vascular network formation was confirmed by immunostaining of TuJ1 and CD31, and HUVECs formed a network around the neurites in the MNS (Figure 3g). The formation of a vascular network of HUVECs provides strong physical support and releases growth factors such as nerve growth factor (NGF), brain‐derived neurotrophic factor (BDNF), and vascular endothelial growth factor (VEGF) that promote the expansion and elongation of neurites.^[^ ^26^ ^]^ Due to the effect of HUVECs, the MNS of groups (ii) and (iii) showed high neurite growth (116.64 ± 12.79 µm and 135.97 ± 18.55 µm, respectively), compared to the group without HUVECs (group (i); 65.77 ± 6.03 µm) (Figure 3h). These results indicated that the introduction of HA@ANA NRs and HUVECs induced the proper alignment and elongation of the MNS to develop a biohybrid robot‐on‐a‐chip.

### 2.3 Generation of a Biohybrid Robot‐on‐a‐Chip

**Figure 4:**
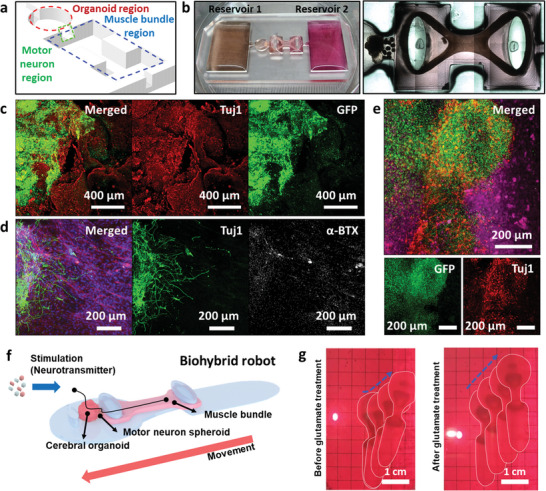
Generation of the human motor system‐based biohybrid robot‐on‐a‐chip. a) Schematic representation of the organoid (red), motor neuron (green), and muscle bundle (blue) regions. b) Optical image of human motor system‐based biohybrid robot‐on‐a‐chip. c) Immunostaining images of representative marker expressions on day 7 of the co‐culturing of cerebral organoid‐multi‐MNSs. Structures of the d) multi‐MNSs‐muscle bundle and e) cerebral organoid‐MNS‐muscle bundle. f) Schematic image of biohybrid robot. g) Snapshot of biohybrid robot locomotion before and after glutamate treatment.

To fabricate the biohybrid robot‐on‐a‐chip, a polymer‐based 3D mold was fabricated using the 3D printing technology (Figure 4a and Figure S6, Supporting Information). First, differentiated multi‐MNSs were mixed with a hybrid hydrogel composed of Matrigel, HA@ANA NRs, and HUVECs and the mixture was seeded in the motor neuron region (Green) in **Figure** **4**a. Next, the cerebral organoid and muscle bundle were each transferred to the polymer‐based 3D mold and connected to the motor neuron region using Matrigel. After coculturing in the maturation medium for cerebral organoids (Contained in reservoir 1) and the maturation medium for multi‐MNSs and muscle bundles (Contained in reservoir 2), the biohybrid robot‐on‐a‐chip was finally fabricated and the connection among the cerebral organoid, multi‐MNSs, and muscle bundle was confirmed through optical imaging (Figure 4b). As shown in Figure S7a,b (Supporting Information), the fabricated biohybrid robot‐on‐a‐chip was showed that each cell was connected after 7 d, and the cerebral organoid was connected to multi‐MNSs by neural cells. Green fluorescent protein (GFP)‐labeled human iPSCs were used to confirm the fusion of the cerebral organoid and multi‐MNSs in the developed biohybrid robot‐on‐a‐chip. Immunostaining of fused cerebral organoid‐multi‐MNSs was performed to confirm the connection between the organoid and motor neuron regions (Figure 4c and Figure S7c, Supporting Information). On day 7 after fusion, it was confirmed that the area covered by GFP gradually increased over time. In addition, the expression of the neural marker Tuj1 was confirmed in the fused cerebral organoid‐multi‐MNSs structure and it was confirmed that the fused structure was completely covered with neural cells. Furthermore, the connection between the multi‐MNS and muscle bundle regions was confirmed using neural cells and the formation of neuromuscular junctions (NMJs).

When NMJs are completely formed, acetylcholine receptors act as finely tuned neurotransmitter‐gated ion channels. Therefore, to confirm the formation of NMJs, immunostaining was performed using alpha‐bungarotoxin (α‐BTX) (NMJs marker, white), F‐actin (Actin filaments, red), and Tuj1 (Neural marker, green) on day 7 of the co‐cultured differentiation of the multi‐MNS and muscle bundle regions (Figure 4d). The elongation of the neurites of multi‐MNSs toward the muscle bundle (Many neurites completely reached the muscle bundle) and the formation of NMJs (α‐BTX) at the end of the neurites was observed. To confirm that the fabricated cerebral organoid‐MNS‐muscle bundle structure was fused as a whole, the overall structure was immunostained (Figure 4e). Immunostaining of the overall structure confirmed that GFP‐labeled cerebral organoid migrated to the MNS and that neurites from the MNSs (TuJ1, red) grew and arrived in the direction of the muscle bundles (F‐actin, purple) and cerebral organoid. Besides, without a polymer‐based 3D mold, developed biohybrid robot showed the forward movement with enhanced speed by glutamate treatment (0.53 ± 0.03 and 1.59 ± 0.32 cm min^−1^ before and after glutamate treatment) (Figure 4f,g and Figure S8a,b and Movie S1, Supporting Information). In addition, the long‐term stable movement of the developed biohybrid robot was investigated for 2 weeks. The developed biohybrid robot exhibited the forward movement for about 10 d with an average movement of 0.73 cm min^−1^, after which the movement gradually became weaker (Figure S8c, Supporting Information). These results confirmed the development of a biohybrid robot‐on‐a‐chip.

### 2.4 Muscle Bundle Movement of a Biohybrid Robot‐on‐a‐Chip

**Figure 5:**
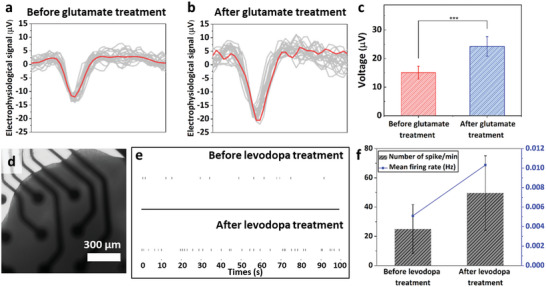
Confirmation of electrophysiological signal analysis of cerebral organoid and midbrain organoid. Electrophysiological signal analysis of cerebral organoid a) before and b) after treatment with 100 × 10^−6^ m of glutamate. c) Quantification of the electrophysiological signals from the cerebral organoid. ***P < 0.001. Error bars correspond to the SD from nineteen measurements. d) Optical image of the PD‐midbrain organoid on the MEA system. e) Spike raster plot of PD‐midbrain organoid before and after treatment with 100 × 10^−6^ m of levodopa. f) Number of spikes and the mean firing rate of PD‐midbrain organoids before and after levodopa treatment.

After the development of a biohybrid robot‐on‐a‐chip, its muscle bundle movement was investigated by evaluating the electrophysiological signal transmission through the connected cerebral organoid and multi‐MNSs. First, the response of the cerebral organoid to a neurotransmitter, glutamate, was investigated through electrophysiological signal analysis (**Figure** **5**a,b). For this, the cerebral organoid was cultured on laminin‐coated MEA system for 3 d, and the electrophysiological signals from the cerebral organoid were measured before and after treatment with 100 × 10^−6^ m of glutamate for 30 min. The electrophysiological signals from the glutamate‐treated cerebral organoid were 1.6 times higher (15.08 ± 2.23 µV) than those from the untreated cerebral organoid (24.26 ± 3.42 µV) (Figure 5c). In addition, the response of the PD‐midbrain organoid treated with levodopa as a dopamine replacement agent for the treatment of PD was confirmed (Figure 5d). To confirm the effect of levodopa to PD‐midbrain organoid, normal midbrain (Control) and PD‐midbrain organoids were generated from healthy and patient‐derived (ND40019) human iPSCs carrying the PD‐associated LRRK2‐G2019S mutation (Figure 5a). Using a low‐attachment round‐bottom 96‐well plate, the healthy and patient‐derived human iPSCs were seeded (5.0 × 10^4^ cells per well) to form a 100 µm size of EBs under maintenance condition (Noggin, SB431542, and CHIR99021). On day 4, the EBs were treated with SB431542, CHIR99021, Noggin, sonic hedgehog (SHH), and fibroblast growth factor 8 for neuroectodermal differentiation and patterning toward midbrain fate. On day 7, the neuroectodermal tissues were embedded into Matrigel for terminal differentiation of midbrain organoid and maturation using BDNF, glial cell line‐derived neurotrophic factor (GDNF), and ascorbic acid for up to 60 d. To confirm the generation of the midbrain organoid, immunostaining analysis of the generated midbrain organoid was performed on day 60 using MAP2, TH, and FOXA2, which are markers for neuronal progenitor cells and neurons (Figure S9b,c, Supporting Information). In addition, to investigate the expression levels of SOX2, NURR1, LMX1A, DAT, and tyrosine hydroxylase (TH), qPCR using midbrain organoid was performed on day 0 and 75 (Figure S9d, Supporting Information). Expression analysis indicated that the SOX2 levels decreased to 0.056 ± 0.036, whereas the NURR1, LMX1A, DAT, and TH levels in the midbrain organoid were higher than those in the undifferentiated human iPSCs (Day 0). Compared with the normal midbrain organoid, the PD‐midbrain organoid was immuno‐stained for synaptophysin (SYP) and postsynaptic density protein 95 (PSD95). Furthermore, as shown in Figure S10a (Supporting Information), the expression level of SYP (red) decreased, and the connection between SYP (Red) and PSD95 (Green) also decreased in the PD‐midbrain organoid. Moreover, the PD‐midbrain organoid showed reduced expression of FOXA2 compared to the normal midbrain organoid. In particular, the expression levels of TH and DAT, which are related to neurons, were reduced compared to the control, and the expression level of SNCA, which makes the alpha‐synuclein protein observed during the early stages of PD, increased (Figure S10b,c, Supporting Information). Furthermore, the electrophysiological activities of the normal midbrain and PD‐midbrain organoid were confirmed using the laminin‐coated MEA system (Figure S11a,b, Supporting Information). Compared to the PD‐midbrain organoid, the normal midbrain organoid (Control) showed a pattern in electrophysiological signals and indicated a higher number of spikes. Furthermore, the number of spikes in normal midbrain organoid was found to be increased by 6.35 times compared to the PD‐midbrain organoid. In addition to the number of spikes, the number of burst increased from 196.5 ± 89.59 to 3.66 ± 0.33 in the case of normal midbrain organiod, and the inter‐burst interval was reduced from 13.83 ± 5.65 to 6.45 ± 2.29 s (Figure S11c,b,d, Supporting Information). In addition, the response of the PD‐midbrain organoid to levodopa was investigated by measuring the electrophysiological signal of the PD‐midbrain organoid before and after treatment with 100 × 10^−6^ m of levodopa for 60 min (Figure 5e). The number of spikes in the PD‐midbrain organoid treated with levodopa increased from 25.0 to 49.7 spikes min^−1^ compared to the normal midbrain organoid before levodopa treatment, and the mean firing rate also increased from 0.005 to 0.013 Hz (Figure 5f).

**Figure 6:**
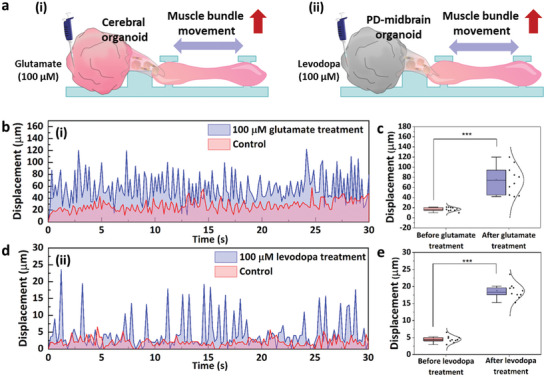
Drug effect evaluation based on muscle bundle movement in the biohybrid robot‐on‐a‐chip. a) Schematic images of the muscle bundle movement of (i) cerebral organoid‐based biohybrid robot‐on‐a‐chip, (ii) PD‐midbrain organoid‐based biohybrid robot‐on‐a‐chip. b,c) Glutamate‐induced muscle bundle movement in the cerebral organoid‐based biohybrid robot‐on‐a‐chip. d,e) Levodopa‐induced muscle bundle movement in the PD‐midbrain organoid‐based biohybrid robot‐on‐a‐chip. ***P < 0.001. Error bars correspond to the SD from ten measurements.

Based on the results which were obtained from individual cerebral organoid or PD‐midbrain organoid by treatment of chemicals (Glutamate and levodopa), the effects of these chemicals to biohybrid robot‐on‐a‐chip were investigated. The glutamate‐enhanced electrophysiological signals from the cerebral organoid were successfully transmitted to the muscle bundle via the connected multi‐MNSs, enhancing the muscle bundle movement (**Figure** **6**a‐i). To confirm this signal transmission, the muscle bundle movement of the fabricated biohybrid robot‐on‐a‐chip was investigated after glutamate treatment. The change in the muscle bundle movement of the biohybrid robot‐on‐a‐chip after glutamate treatment was tracked using a microscope. As shown in Movie S2 (Supporting Information), the contraction of muscle bundles before (Control) and after treatment with 100 × 10^−6^ m of glutamate was recorded for 30 min. The muscle bundle displacement increased to 74.81 ± 27.79 µm due to the glutamate‐induced enhancement of the electrophysiological activity of the cerebral organoid; thus, a 4.41 times improvement in movement compared to that before glutamate treatment was recorded (Figure 6b,c). Furthermore, since the developed biohybrid robot‐on‐a‐chip mimics the human motor system in which electrophysiological signals from the CNS generate skeletal muscle movements through motor neurons, we introduced tetrodotoxin (TTX), a neurotoxin, into the biohybrid robot‐on‐a‐chip to block the transmission of electrophysiological signals from cerebral organoid and confirmed the changes in muscle bundle movements accordingly. As shown in Figure S12 (Supporting Information), we confirmed that the movement of the muscle bundle was reduced from 9.51 ± 0.49 µm to 30.55 ± 1.18 µm when treated with 1 × 10^−6^ m of TTX, and the movement was restored when TTX was washed away. In addition, a PD‐midbrain organoid‐based biohybrid robot‐on‐a‐chip was fabricated using PD‐midbrain organoid instead of cerebral organoid to evaluate the effect of a drug (Levodopa) on PD that causes muscle malfunction. The effects of levodopa on PD were confirmed using a biohybrid robot‐on‐a‐chip by measuring muscle movement as well as electrophysiological signals (Figure 6a‐ii). Like the cerebral organoid‐based biohybrid robot‐on‐a‐chip, 100 × 10^−6^ m of levodopa was added to reservoir 1 of the PD‐midbrain organoid‐based biohybrid robot‐on‐a‐chip. As shown in Movie S3 (Supporting Information), the movements of muscle bundles before (Control) and after treatment with 100 × 10^−6^ m of levodopa for 10 h were recorded. The muscle bundle displacement increased to 18.67 ± 2.25 µm after levodopa treatment; thus, a 4.15 times improvement in movement compared to that before levodopa treatment was observed (Figure 6d,e). In addition to measuring the muscle bundle movement, we measured changes in the contraction force of the muscle bundle of the PD‐midbrain organoid‐based biohybrid robot‐on‐a‐chip by levodopa treatment. As shown in Figure S12a,b (Supporting Information), a pillar composed of Ecoflex was fabricated for the measurement, and the displacement of the edge of the pillar was recorded using a microscope to calculate the contraction force. The measured contraction force of the muscle bundle increased by 1.90 times from 15.25 ± 1.02 µN to 29.05 ± 1.83 µN by levodopa treatment (Figure S13c,d, Supporting Information). Thus, the effect of a drug (Levodopa) on a neurodegenerative disease (PD) was successfully evaluated using the developed human motor system‐based biohybrid robot‐on‐a‐chip.

## 3 Conclusion

Traditional methods for promoting muscle movement to drive the biohybrid robot, such as spontaneous muscle movement or electrical/optical muscle stimulation, require complex processes, including genetic manipulation and the introduction of external electrodes for stimulation.^[^ ^27^ ^]^ In addition, most biohybrid robots driven by muscle cells and motor neurons cannot mimic the human motor system to simulate a neurodegenerative disease related to CNS and muscle movement due to absence of brain function.^[^ ^10^ ^]^ In the case of OoCs, the CNS model has been developed, but it is difficult to study neurodegenerative diseases because the muscles are not connected to the CNS model. To address these limitations, it is essential to connect the brain and muscles and enable the control of muscle cells by the brain or neural signals. Overcoming these limitations will provide a promising strategy for the application of the biohybrid robot to study neurodegenerative diseases. To achieve this, a brain organoid, which can accurately simulate muscle movement through the connected motor neuron similar to the human motor system, was used. However, connecting the brain organoid and motor neurons to control the movement of the muscles remains a challenge. In previous studies, we reported that the brain–spinal cord assembloid is capable of transmitting electrophysiological signals from the cerebral organoid to the connected MNS.^[^ ^28^ ^]^ In addition, we demonstrated that the 3D NMJ system composed of MNS and muscle bundle can successfully transmit signals between the MNS and muscle bundle.^[^ ^29^ ^]^ However, so far, no study has demonstrated an electrophysiological signal transmission from the brain organoid to the muscle bundle via the motor neuron for muscle movement, which is essential for evaluating the effects of drugs on neurodegenerative diseases.

In this study, the human motor system‐based biohybrid robot‐on‐a‐chip composed of a brain organoid, multi‐MNSs, and muscle bundle on the solid substrate was developed for the first time by connecting the cerebral organoid with muscle bundle using multi‐MNSs with HUVECs and HA@ANA NR to evaluate the effects of drugs on neurodegenerative diseases. The HA@ANA NR allowed the motor neurons to be oriented while simultaneously allowing the neural population of the multi‐MNSs from HUVECs to efficiently form a neural network connecting the cerebral organoid and muscle bundle. The muscle bundle movement increased with the glutamate treatment‐induced increase in electrophysiological signals from the cerebral organoid, and the muscle bundle displacement increased to 74.81 ± 27.79 µm, which was 4.41 times higher than that observed before glutamate treatment. The results presented here demonstrated the establishment of a novel strategy for evaluating the effects of drugs by measuring muscle bundle movement through cerebral organoid regulation. In addition, the PD patient‐derived midbrain organoid‐based biohybrid robot‐on‐a‐chip showed that the muscle bundle displacement increased from 4.5 ± 0.99 µm to 18.67 ± 2.25 µm in response to levodopa. Even though only the muscle bundle movement by unlearned cerebral organoid and midbrain organoid was confirmed to evaluate the effect of a drug on neurodegenerative disease in this study, this approach can be used for more accurate drug evaluation by introducing cerebral organoids with a learning function using a neural network. Moreover, the introduction of sensing organoids such as an eye organoid into the developed biohybrid robot‐on‐a‐chip can lead to the development of biohybrid robot‐on‐a‐chip with learning and sensing functions, enabling a conscious response. In conclusion, the developed human motor system‐based biohybrid robot‐on‐a‐chip can serve as a standard biohybrid robot model for drug evaluation.

## Conflict of Interest

The authors declare no conflict of interest.

## Supporting information

Supporting Information

Supplemental Movie 1

Supplemental Movie 2

Supplemental Movie 3

## Data Availability

Research data are not shared.
