# Supplementary material for: Human Motor System‐Based Biohybrid Robot‐On‐a‐Chip for Drug Evaluation of Neurodegenerative Disease
Source: Adv Sci (Weinh). 2023 Nov 30;11(4):2305371. doi: 10.1002/advs.202305371 (PMC10811491; doi:10.1002/advs.202305371)
Supplement: Supplementary file 1 — Supporting Information [file ADVS-11-2305371-s004.pdf]

ADVANCED SCIENCE
Open Access

Supporting Information

for *Adv. Sci.*, DOI 10.1002/advs.202305371

Human Motor System-Based Biohybrid Robot-On-a-Chip for Drug Evaluation of Neurodegenerative Disease

Minkyu Shin, Taehyeong Ha, Joungpyo Lim, Joohyun An, Geunyoung Beak, Jin-Ha Choi, Ambrose Ashwin Melvin, Jinho Yoon and Jeong-Woo Choi**

SUPPORTING INFORMATION

Human motor system-based biohybrid robot-on-a-chip for drug evaluation of neurodegenerative disease

Minkyu Shin[1,†], Taehyeong Ha[1,†], Joungpyo Lim[1], Joohyun An[1], Geun Young Beak[1], Jin-Ha Choi[2], Ambrose Ashwin Melvin[1], Jinho Yoon[3,4], Jeong-Woo Choi[1,*]*

[1]Dr. M. Shin, Dr. T. Ha, Mr. J. Lim, Ms. J. An, Mr. G. Y. Beak, Dr. A. A. Melvin, Prof. J. -W. Choi
Department of Chemical & Biomolecular Engineering, Sogang University, 35 Baekbeom-ro, Mapo-gu, Seoul 04107, Republic of Korea
E-mail: jwchoi@sogang.ac.kr
res

[2]Prof. J.-H. Choi
School of Chemical Engineering, Jeonbuk National University, 567 Baekje-daero, Deokjin-gu, Jeonju-si, Jeollabuk-do 54896, Republic of Korea

[3]Prof. J. Yoon
Department of Biomedical-Chemical Engineering, The Catholic University of Korea, 43 Jibong-ro, Wonmi-gu, Bucheon-si, Gyeonggi-do 14662, Republic of Korea

[4]Prof. J. Yoon
Department of Biotechnology, The Catholic University of Korea, 43 Jibong-ro, Wonmi-gu, Bucheon-si, Gyeonggi-do 14662, Republic of Korea
E-mail: jyoon@catholic.ac.kr

† These authors contributed equally to this work as the first authors.

TABLE OF CONTENTS FOR SUPPORTING INFORMATION

A. METHODS

B. SUPPLEMENTARY FIGURES

**Figure S1**	Generation of cerebral organoid. a) Time-dependent morphological changes in the cerebral organoid. b) Cerebral organoid on day 60.
**Figure S2**	Electrophysiological signals from the cerebral organoid. Spike raster plot on a) day 30 and b) day 90. c,d, Electrophysiological quantification of the number of (c) spikes and (d) bursts. **P < 0.01. Error bars correspond to the standard deviation (SD) from four measurements.
**Figure S3**	Confirmation of ANA NRs synthesis. a) Schematic representation of the synthesis process of HA@ANA NRs. b,c) SEM image of ANA NRs and c,d) EDS and EDS mapping of ANA NRs.
**Figure S4**	Confirmation of surface modification of HA@ANA NR. a) TEM and EDS mapping image of HA@ANA NRs. b) Zeta potential analysis of ANA NRs and HA@ANA NR.
**Figure S5**	Confirmation of directionality of the HA@ANA NR. Opitcal images of a) before alignment and b) after alignment. c) quarter-circle graphs showing the directionality of HA@ANA NRs
**Figure S6**	Fabrication of the polymer mold for the biohybrid robot-on-a-chip. a) Schematic representation of the fabrication process of polymer mold for the biohybrid robot-on-a-chip. Optical images of the b) 3D print and c) PDMS molds.
**Figure S7**	Generation of the midbrain organoid. a) Optical image of human motor system-based biohybrid robot-on-a-chip. b) Optical image and c) immunostaining image of the day 7 of the co-culturing of cerebral organoid–multi-MNSs.
**Figure S8**	Confirmation of forward movement of biohybrid robot. a) Displacement and b) velocity of the biohybrid robot. c) long-term mobility capabilities of the biohybrid robot.*P < 0.05. Error bars correspond to the SD from four measurements.
**Figure S9**	Generation of the midbrain organoid. a) Time-dependent morphological changes in the midbrain organoid. b,c) Immunostaining

images of the midbrain organoid. d) Normalized expression levels of SOX2, NURR1, LMX1A, DAT, and TH in the midbrain organoid. *P < 0.05, **P < 0.01, and ***P < 0.001. Error bars correspond to the SD from four measurements.

Figure S10 Confirmation of PD-midbrain organoid. a,b) Immunostaining images of normal midbrain and PD-midbrain organoids. c) Normalized expression levels of TH, DAT, and SNCA in normal midbrain and PD-midbrain organoids. *P < 0.05, **P < 0.01, and ***P < 0.001. Error bars correspond to the SD from four measurements.

Figure S11 Electrophysiological signals in the midbrain organoid. Spike raster plot of a) normal midbrain and b) PD-midbrain organoids. Electrophysiological quantification of the number of c) number of spikes and d) number of bursts, and e) inter-burst interval of normal midbrain organoid and PD-midbrain organoid. **P < 0.01. Error bars correspond to the SD from four measurements.

Figure S12 Confirmation of correation between the muscle bundle movement and CNS neural pathway. a) Time-dependent morphological changes in the midbrain organoid. a,b) TTX-induced muscle bundle movement in the cerebral organoid-based biohybrid robot-on-a-chip. ***P < 0.001. Error bars correspond to the SD from four measurements.

Figure S13 Drug evaluation based on the contraction force of muscle bundle. a) Schematic images of the measurement of muscle bundle contraction using the edge of pillar. b) Optical image of displacement of the edge of pillar by the muscle bundle contraction. c,d) Levodopa-induced muscle bundle contraction in the PD-midbrain organoid-based biohybrid robot-on-a-chip. ***P < 0.001. Error bars correspond to the SD from four measurements.

Table S1 List primers for qPCR.

Movie S1 Biohybrid robot.

Movie S2 Muscle bundle movement of cerebral organoid-based biohybrid robot-on-a-chip induced by the glutamate.

Movie S3 Muscle bundle movement of PD-midbrain organoid-based biohybrid robot-on-a-chip induced by the levodopa.

A. METHODS:

Generation of Cerebral Organoid: Human iPSCs were cultured on iMatrix-511 silk-coated culture dish in StemFit Basic04 (Ajimoto). The EBs were generated from iPSCs using ultra-low attachment round-bottom 96-well plates (SPL Life Science) for 24 h by seeding the 1.0 $\times 10^4$ cells per well with StemFit Basic04 by addition of 50 μM of Rho-associated protein kinase (ROCK) inhibitor (Y-27632 dihydrochloride, Biogems) and 4 ng/mL of basic fibroblast growth factor (bFGF) (Peprotech). After 24 h from seeding, EBs were formed, and the medium was changed by fresh StemFit Basic04 medium with 4 ng/mL of bFGF and 50 μM of ROCK inhibitor. After 2 days from EB generation (day 4), the medium was changed with fresh StemFit Basic04 medium containing bFGF with 2 ng/mL. On day 5, EBs were transferred to 60 mm ultra-low attachment Petri dish. From day 5 to day 9, the medium was changed every two days with DMEM/F-12 (WELGENE) supplemented with 1 × N2 supplement (Thermo Fisher Scientific), 1 × GlutaMAX supplement (Gibco), 1 × MEM-NEAA solution (Sigma-Aldrich), and 1 µg/mL of heparin (Sigma-Aldrich) for neuroepithelium-like tissue formation. On day 9, each neuroepithelial tissue was embedded with Matrigel droplet (5 μL) on a sterilized 60 mm dish with embedding sheet (STEMCELL Technologies) and placed in a 37 ℃ incubator for 30 min to achieve the polymerization. After the polymerization of Matrigel (Corning), addition of 8 mL of DMEM/F-12 and Neurobasal media (Thermo Fisher Scientific) prepared with 1:1 ratio, containing N2 supplement of 1:200 (v/v), B-27 supplement of 1:100 (v/v) without vitamin A (Gibco), GlutaMAX supplement of 1:100 (v/v), MEM-NEAA solution of 1:200 (v/v), 50 μM of 2-mercaptoethanol (Gibco), and 2.5 µg/mL of insulin (Sigma-Aldrich) was conducted. Next, addition of 100 µg/mL of streptomycin (Gibco) and 100 U/mL of penicillin was done sequentially. The Matrigel were detached from the embedding sheet and the medium was changed every 2 days. On day 13, the dish prepared with cerebral tissues was placed on orbital shaker and the maturation medium was changed every 3 days with DMEM/F-12 and Neurobasal media prepared with 1:1 ratio which containted 1:200 (v/v) N2 supplement, 1:200 (v/v) MEM-NEAA solution, 1:100 (v/v) GlutaMAX supplement, 2.5 µg/mL of insulin, 50 μM of 2-mercaptoethanol, and 1:100 (v/v) B-27 supplement (Gibco). Next, addition of streptomycin (Gibco) with 100 µg/mL and penicillin with 100 U/mL was done. The immunostaining, qPCR, and electrophysiological analysis of cerebral organoids were conducted on days 35-60.

Generation of MNS: To maintain Human NSCs, they were seeded on laminin-coated tissue culture plate with StemPro Neural supplement (Gibco) (20 μL/mL), bFGF (20 ng/mL), KnockOut DMEM/F-12 medium (Gibco), ascorbic acid (200 μM, Sigma-Aldrich), GlutaMAX supplement (2 mM), heparin (6 units/mL), and EGF (20 ng/mL, Peprotech). By seeding the NSCs (5.0 $\times 10^4$ cells per well) on 96-well plates with round-bottomed ultra-low attachment, neural spheroids were produced. After 24 h from NSCs seeding, the mixture, composed of StemPro hESC medium (Gibco), 10 ng/mL of activating A (Peprotech), 50 μM of retinoic acid

(Sigma-Aldrich), 8 ng/mL of bFGF, and 200 ng/mL of sonic hedgehog (Peprotech), replaced a culture medium to induce the motor neuron differentiation. On 20 days After, the mixture composed of StemPro hESC medium (Gibco), 10 ng/mL of GDNF (Peprotech), 10 ng/mL of BDNF (Peprotech) was treated to maturate motor neuron for 8 days.

Generation of Muscle Bundle: Prior to the muscle bundle generation, C2C12 cells were maintained using DMEM cell culture media containing antibiotics (penicillin with 100 U/mL and streptomycin with 100 μg/mL) and 10% FBS (YoungIn Frontier). To fabricate the muscle bundle, 340 μL of C2C12 cells (5×10^6 cells) were mixed with 300 μL of Matrigel, 250 μL of fibrinogen (16 mg/mL concentration) obtained from Sigma-Aldrich, 10 μL of thrombin, and 100 μL of DMEM. Then, 200 μL of prepared mixture was dropped into the PDMS mold and placed in a 37 ℃ incubator for 30 min for polymerization. To induce the differentiation of prepared muscle bundle, the DMEM containing 1 mg/mL of aminocaproic acid (Sigma-Aldrich), 1 ng/mL of insulin growth factor-1 (Sigma-Aldrich) and 1:50 horse serum (Gibco) was used as a differentiation medium. Next, addition of penicillin with 100 U/mL and streptomycin with 100 μg/mL was conducted, and a differentiation medium was changed every 2 days for about 2 weeks.

Synthesis of hyaluronic acid modified Au-Ni-Au nanorod: To synthesize HA@ANA NR, first, thiolated HA was prepared. For this, 100 mg of HA (Sigma-Aldrich) and 60 mg of cysteamine dihydrochloride (Sigma-Aldrich) were stirred for 2 h with 0.4 M NaCl and 10 mL of 0.1 M boric acid buffer under pH 8.5. 200 nM of sodium cyanoborohydride (Sigma-Aldrich) was added to the prepared buffer and the mixture was stored at 40 °C for 5 days. 100 mM of dithiothreitol (DTT) was added and reacted for 12 h to introduce thiol groups to HA. The reacted mixture was dialyzed by more than 100 mM NaCl for 2 days. Sequentially, it was treated by 25% ethanol for 1 day, and by distilled water for 1 day for removing unreacted chemical molecules. Next the mixture was dried completely for 3 days to obtain thiolated HA. To synthesize of ANA NR, as a conductive layer, a 300 nm thickness of Ag layer was prepared on one side of the AAO template (Citiva) by physical vapor deposition. After contacting the aluminum foil with the surface of the AAO template deposited with Ag, electrochemical deposition was performed using a three-electrode system. For stable electrochemical deposition on the AAO template, a commercial Ag plating solution (Alfa Aesar) was pre-deposited at -0.95 V. To synthesize ANA NRs, Au and Ni components were successively deposited with commercial plating solutions (Alfa Aesar) at -0.95 V (vs Ag/AgCl). Specifically, 1 Coulomb of Au, 0.5 Coulomb of Ni, and 1 Coulomb of Au were deposited sequentially. Next, The Ag layer on the AAO template was etched by nitric acid and the AAO template was degraded by 3 M NaOH completely. Then, the remained samples were rinsed by using distilled water. The composition of the synthesized ANA NRs was characterized by SEM images. After synthesis of ANA NRs, 1 mg of ANA NRs were mixed with 10 mL of

thiol-modified HA (0.4 mg/mL) for 24 h and HA@ANA NRs were finally prepared and collected magnetically using Ni component in HA@ANA NRs.

Generation of biohybrid robot-on-a-chip: To fabricate the biohybrid robot-on-a-chip, the mold of a biohybrid robot-on-a-chip was designed using 3D MAX and fabricated by Stereolithography (SLA) 3D printer (Formlabs) with photocurable resin. Then, the polydimethylsiloxane (PDMS) (Dow Corning) was poured inside the prepared mold and cured at 70 ℃ for 4 h. The fabricated mold for biohybrid robot-on-a-chip was washed with ethanol and distilled water. First, differentiated multi-MNSs were mixed with hybrid hydrogel composed of 60 µL of Matrigel, 20 µL of HA@ANA NRs (0.5 mg/mL), 68 µL of 1 $\times 10^6$ HUVECs (Lonza), and 50 µL of fibrinogen (16 mg/mL). Then, the 10 µL of hybrid hydrogel containing the differentiated multi-MNSs was seeded into the motor neuron region (green) and placed in a 37 ℃ incubator for 30 min (**Figure 4a**). After the polymerization of hybrid hydrogel containing the differentiated multi-MNSs, the brain organoids and muscle bundle were positioned in the organoid region (red) and muscle bundle region (blue), respectively. For connections among the cerebral organoid, multi-MNSs, and muscle bundle, 10 µL of Matrigel containing HUVECs was added to both connections between the cerebral organoid and multi-MNSs, and between multi-MNSs and muscle bundle. The biohybrid robot-on-a-chip was fabricated by co-culturing cerebral organoid maturation medium for brain organoid in reservoir 1 and medium for multi-MNSs and muscle bundle (muscle bundle differentiation medium supplemented with 10 ng/mL of GNDF and 10 ng/mL of BDNF) in reservoir 2 for 7 days. In addition, the medium inside the reservoir was replaced by exchanging half of the medium every day to maintain the viability of cells composing the biohybrid robot on-a-chip. For confirmation of biohybrid robot movement, the connection of each cell was implemented on a mixture of a 5:1 ratio of Ecoflex 00-30/polydimethylsiloxane (PDMS)-based biohybrid robot structure using the same fabrication method as the biohybrid robot-on-a-chip, and the driving experiment was performed in co-culture media (Cerebral organoid maturation medium + muscle bundle differentiation medium supplemented with 10 ng/mL of GNDF and 10 ng/mL of BDNF).

Tissue clearing and immunohistochemistry: The Clear, Unobstructed Brain Imaging Cocktails (CUBIC) were used to clear the brain organoid. For this, 4% paraformaldehyde (Biosesang) was treated to brain organoids for fixation, and then, the fixed brain organoids were then washed with DPBS and immersed in CUBIC-1 solution consisting of 10% (w/w) of urea, 5% (w/w) of NNNN-tetrakis (2-HP) ethylenediamine, distilled water, and Triton X-100 for 3 days. Then, brain organoids were washed by using DPBS. Following the process of clearing, primary antibodies: Tuj1 (1:250, Abcam, EP1569Y), anti-SOX2 (1:50, Abcam,

ab93689), anti-PAX6(1:50, Santa Cruz Biotechnology, sc-81649), and anti-S100 beta (1:100, Abcam, ab52642), were treated for 3 days at 25 ℃. After that, brain organoids were washed by using DPBS, and they were treated with secondary antibodies [(FITC-modified IgG (1: 100) and Texas Red-modified IgG (1:100)] for 2 days, and a nuclei of brain organoid was stained by using Hoechst (3 μg/mL) for 3 min. Next, the prepared brain organoids were stored in CUBIC-2 solution composed of 10% (w/w) triethanolamine, 50% (w/w) sucrose, distilled water, and 25% (w/w) urea for 1 day to become transparent. Immunostaining analyses were conducted by using a confocal microscope (Carl Zeiss, Germany).

For immunostaining analysis, the MNSs and muscle bundle were fixed by using 4% (w/v) formaldehyde for 30 min at 25 ℃. After three washes with DPBS, the fixed samples were immersed in the 0.2% Triton-X for permeabilization for 60 min and blocked by 2% bovine serum albumin (BSA) for 30 min. Prepared samples were incubated overnight at 4 °C with primary antibodies. As primary antibodies, Alexa Flour 488 alpha-bungarotoxin (α-BTX) (1:500, Invitrogen), mouse monoclonal anti-islet1 (islet1) (1:500, Abcam, 1B1), the rabbit monoclonal anti-neuron-specific class III β-tubulin (Tuj1) (1:1000, Abcam, EP1569Y), sarcomeric α-actinin (1:100, Abcam), MHC (1:100, Santa Cruz Biotechnology), and Alexa Flour 546 Phalloidin (F-action) (1:1000, Invitrogen) were used. After culturing with primary antibodies, samples were cleaned by DPBS and treated with secondary antibodies. Immunostaining analyses were done by using a confocal microscopy (LSM 710, Carl Zeiss, Jena, Germany).

qPCR analysis: To verify the differentiation of cells into the brain organoids, MNSs, and muscle bundle, qPCR analysis was conducted. First, the genomic RNA was isolated from each sample using TRIzol reagent, and then the reverse transcription step was conducted using a AccuPower CyclerScript RT PreMix (Bioneer). Next, using a Exicycler 96 system (Bioneer) with AccuPower Taq PCR PreMix (Bioneer), qPCR analysis was performed. The expression level of mRNA (GAPDH, a housekeeping gene) was used as the standard for normalization of all obtained data. The information of primers used in qPCR analysis is provided in Table S1.

Recording of electrophysiological signals: The electrophysiological signals of cerebral organoid, midbrain organoid, and PD-midbrain organoid were recorded by MEA system obtained from Axion BioSystems. The 16 electrode arrays containing MEA system was precoated by 0.1% poly(ethyleneimine) (PEI) 60 min at 37 ℃. Then, laminin was coated on the prepared MEA plate to increase the cell attachemnt for 3 h at 37 ℃. The cerebral organoid, midbrain organoid, and PD-midbrain organoid were cultured on the laminin-coated MEA system for 3 days at 37 ℃ with each culture medium and their electrophysiological signals were recorded under 12.5 kHz for 5 min. During the signal recording, as an optimal condition, Butterworth bandpass filter (200 Hz to 3,000 Hz cutoff frequency) and a threshold of 6 × standard deviation (SD) were

established to reduce the false positive signals using Axion Integrated Studio program. To analyze the obtained spike raster graphs, the Neural Metric Tool (Axion BioSystems) was utilized.

B. SUPPLEMENTARY FIGURES AND TABLES:

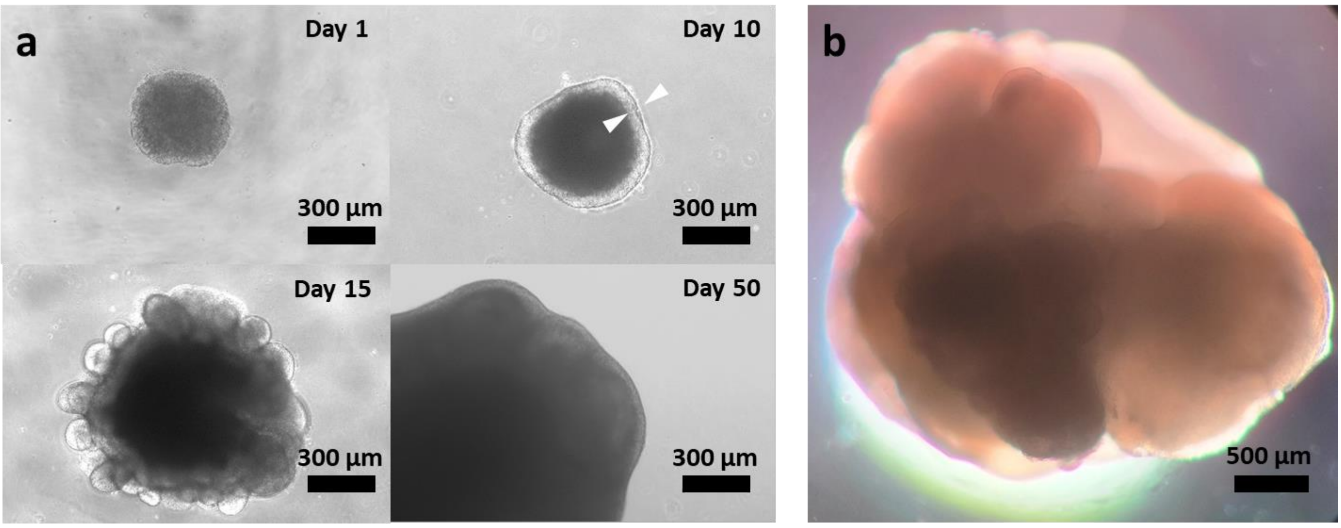

Figure S1. Generation of cerebral organoid. a) Time-dependent morphological changes in the cerebral organoid. b) Cerebral organoid on day 60.

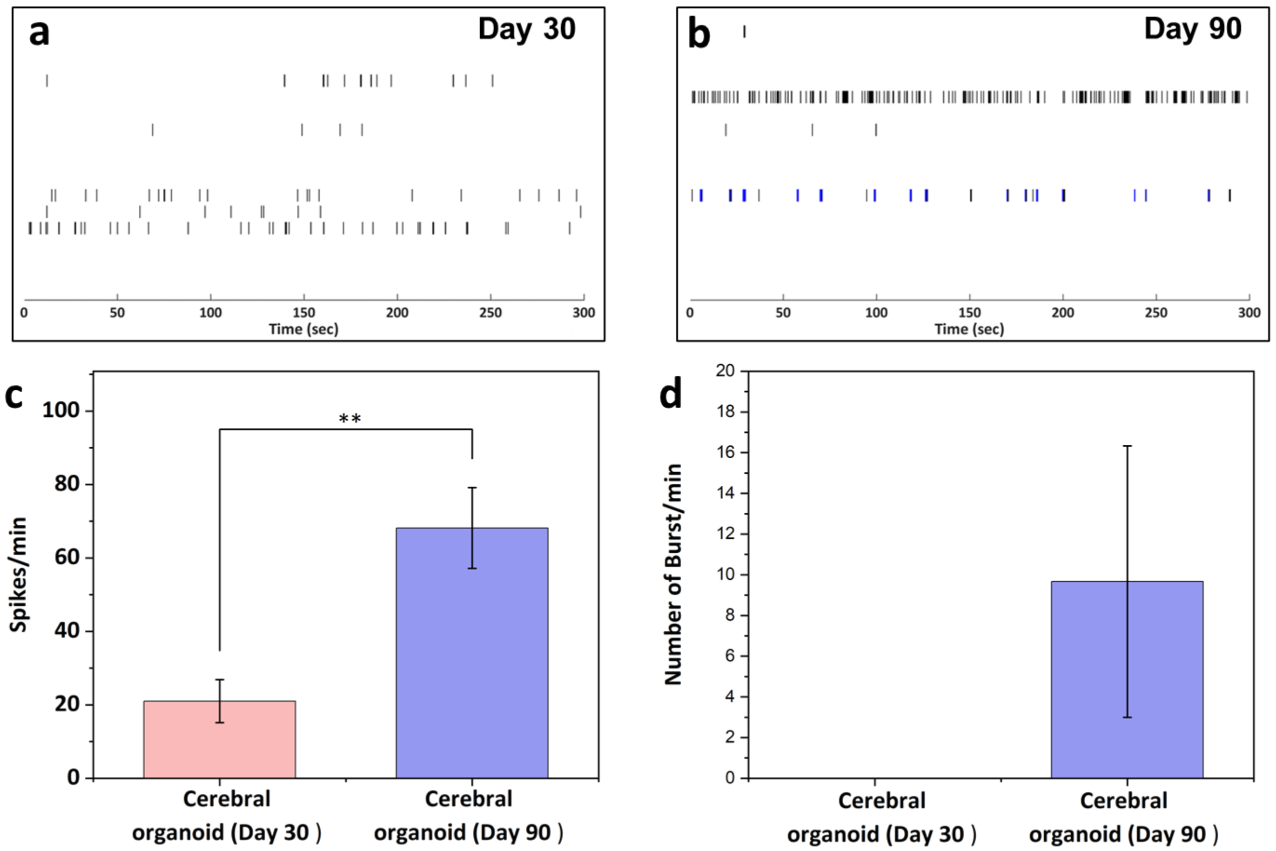

Figure S2. Electrophysiological signals from the cerebral organoid. Spike raster plot on a) day 30 and b) day 90. Electrophysiological quantification of the number of c) spikes and d) bursts. **P < 0.01. Error bars correspond to the standard deviation (SD) from four measurements.

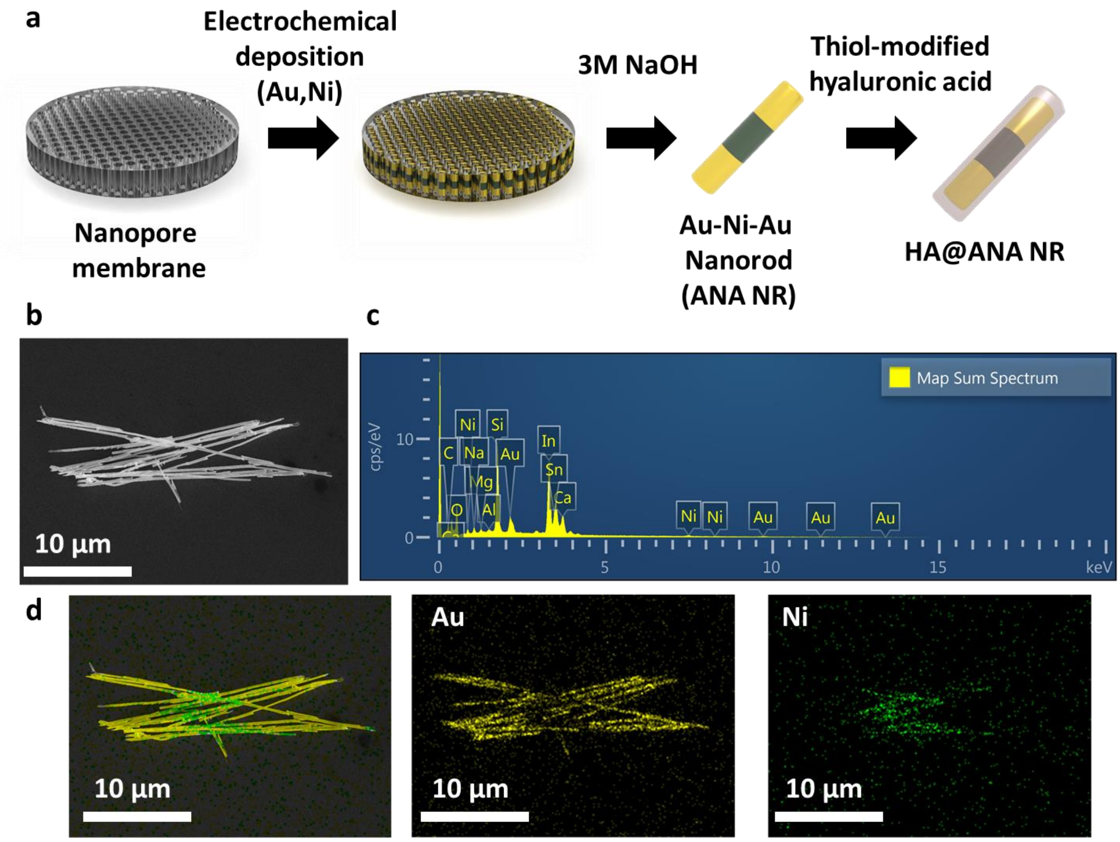

Figure S3. Synthesis of ANA NRs and the confirmation of surface modification. a) Schematic representation of the synthesis process of HA@ANA NRs. b) SEM image of ANA NRs and c,d) EDS and EDS mapping of ANA NRs.

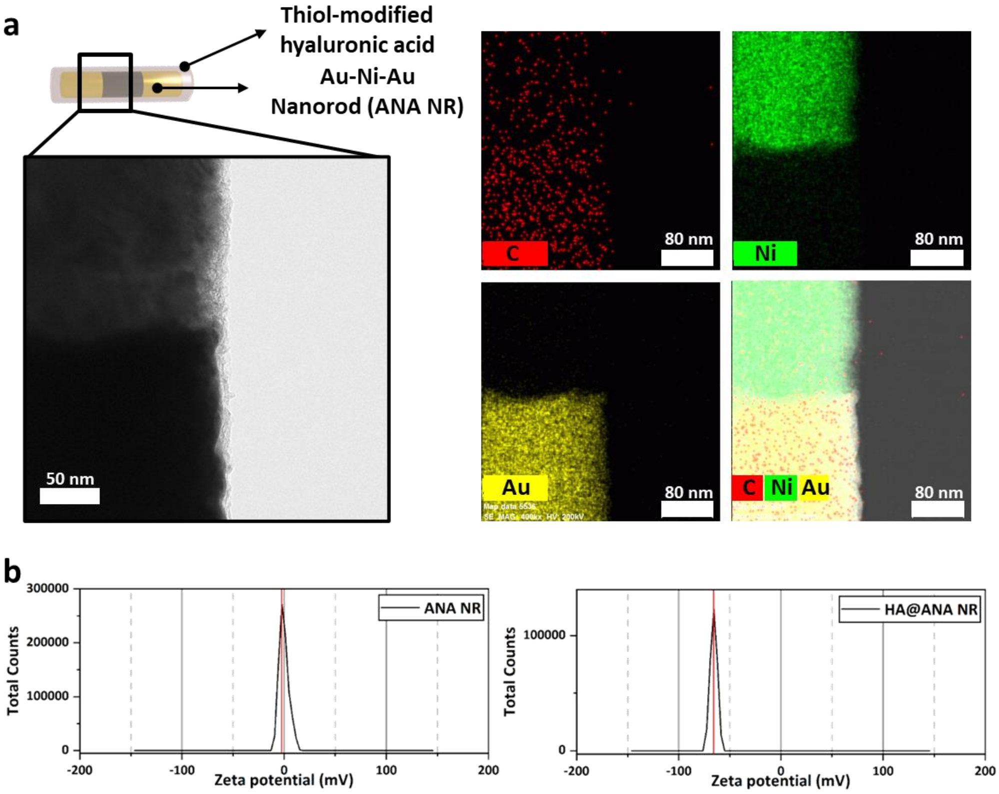

Figure S4. Confirmation of surface modification of HA@ANA NR. a) TEM and EDS mapping image of HA@ANA NRs. b) Zeta potential analysis of ANA NRs and HA@ANA NR.

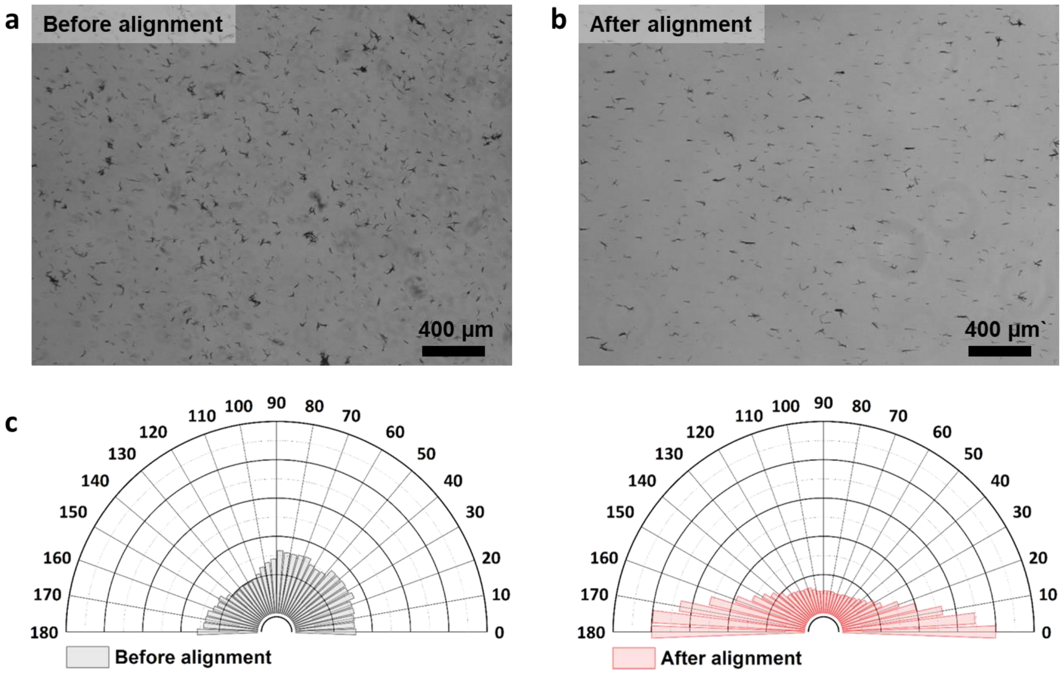

Figure S5. Confirmation of directionality of the HA@ANA NR. Opitcal images of a) before alignment and b) after alignment. c) quarter-circle graphs showing the directionality of HA@ANA NRs

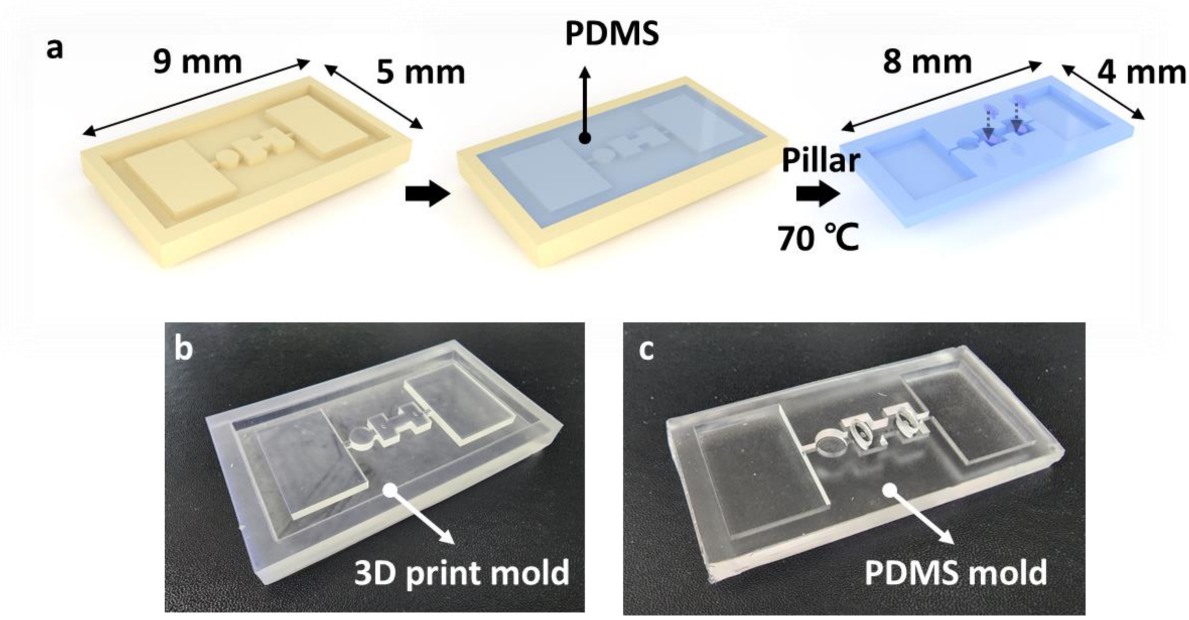

Figure S6. Fabrication of the polymer mold for the biohybrid robot-on-a-chip. a) Schematic representation of the fabrication process of polymer mold for the biohybrid robot-on-a-chip. Optical images of the b) 3D print and c) PDMS molds.

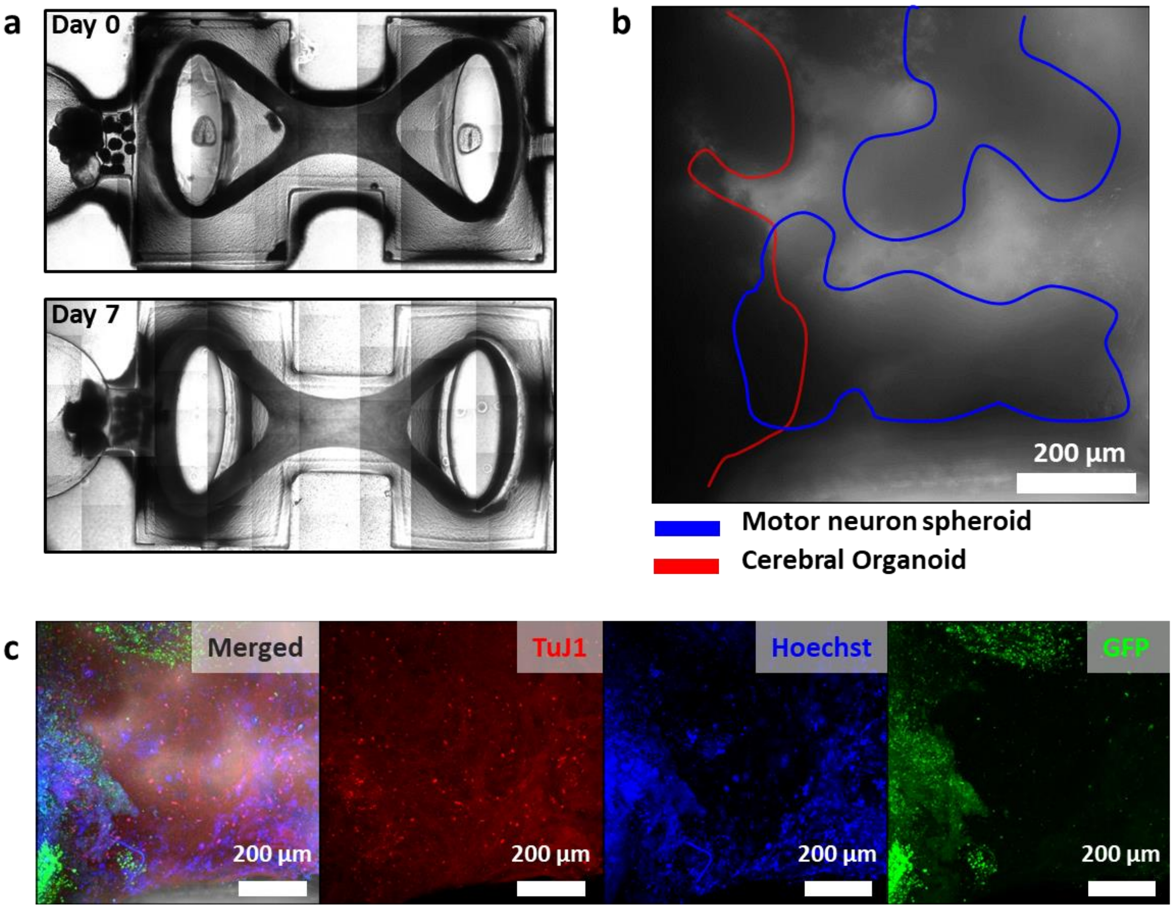

Figure S7. Generation of the midbrain organoid. a) Optical image of human motor system-based biohybrid robot-on-a-chip. b) Optical image and c) immunostaining image of the day 7 of the co-culturing of cerebral organoid–multi-MNSs.

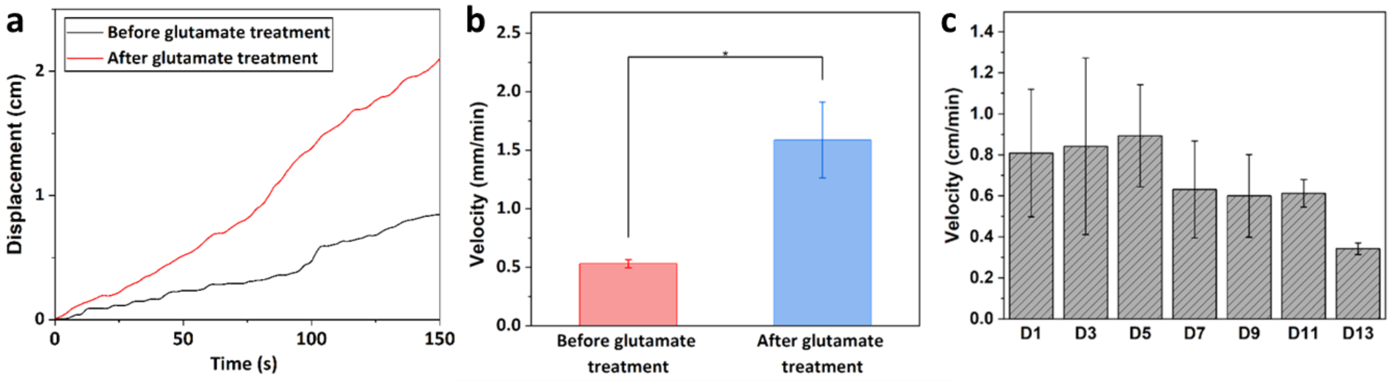

Figure S8. Confirmation of forward movement of biohybrid robot. a) Displacement and b) velocity of the biohybrid robot. c) long-term mobility capabilities of the biohybrid robot. *P < 0.05. Error bars correspond to the SD from four measurements.

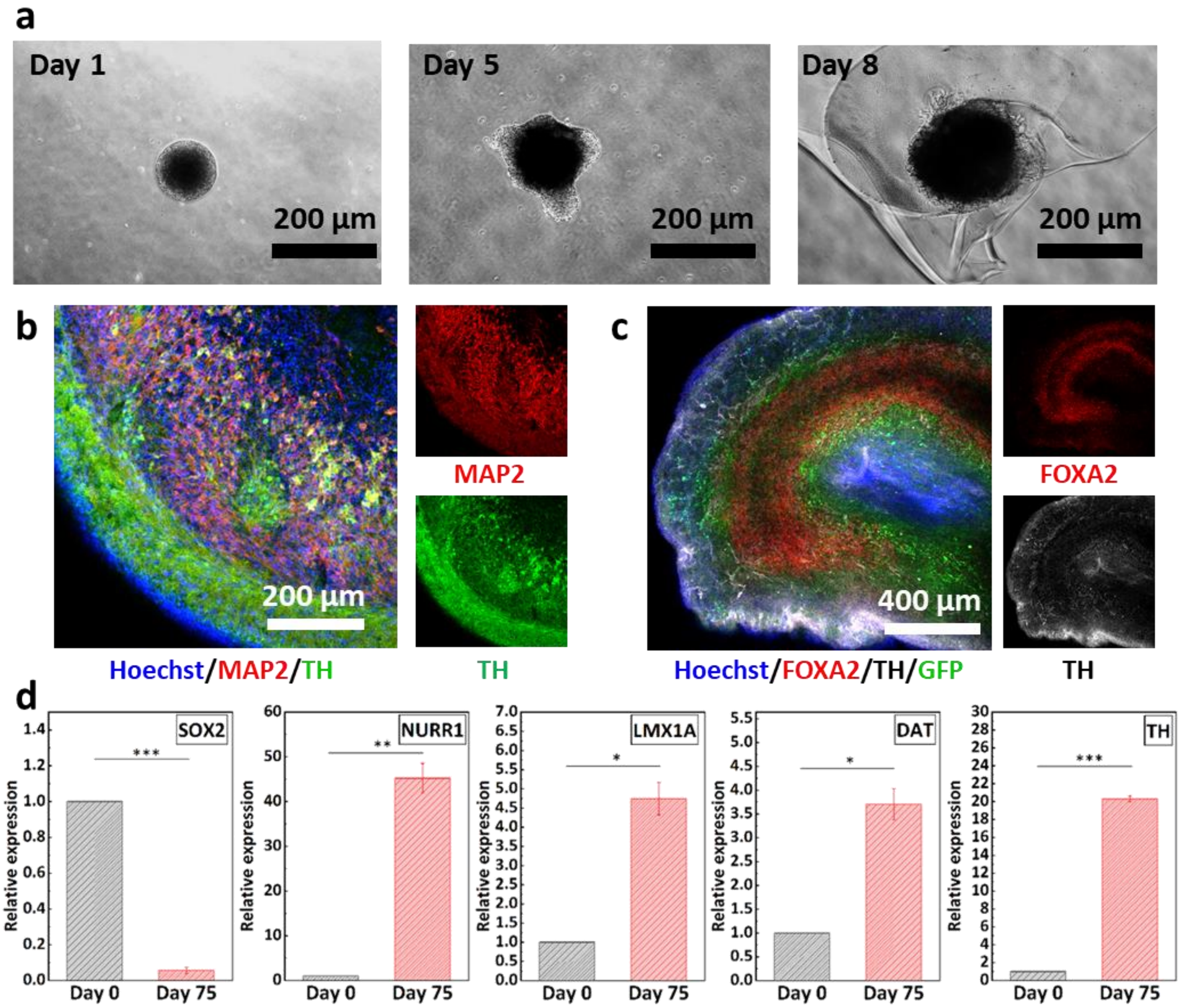

Figure S9. Generation of the midbrain organoid. a) Time-dependent morphological changes in the midbrain organoid. b,c) Immunostaining images of the midbrain organoid. d) Normalized expression levels of SOX2, NURR1, LMX1A, DAT, and TH in the midbrain organoid. *P < 0.05, **P < 0.01, and ***P < 0.001. Error bars correspond to the SD from four measurements.

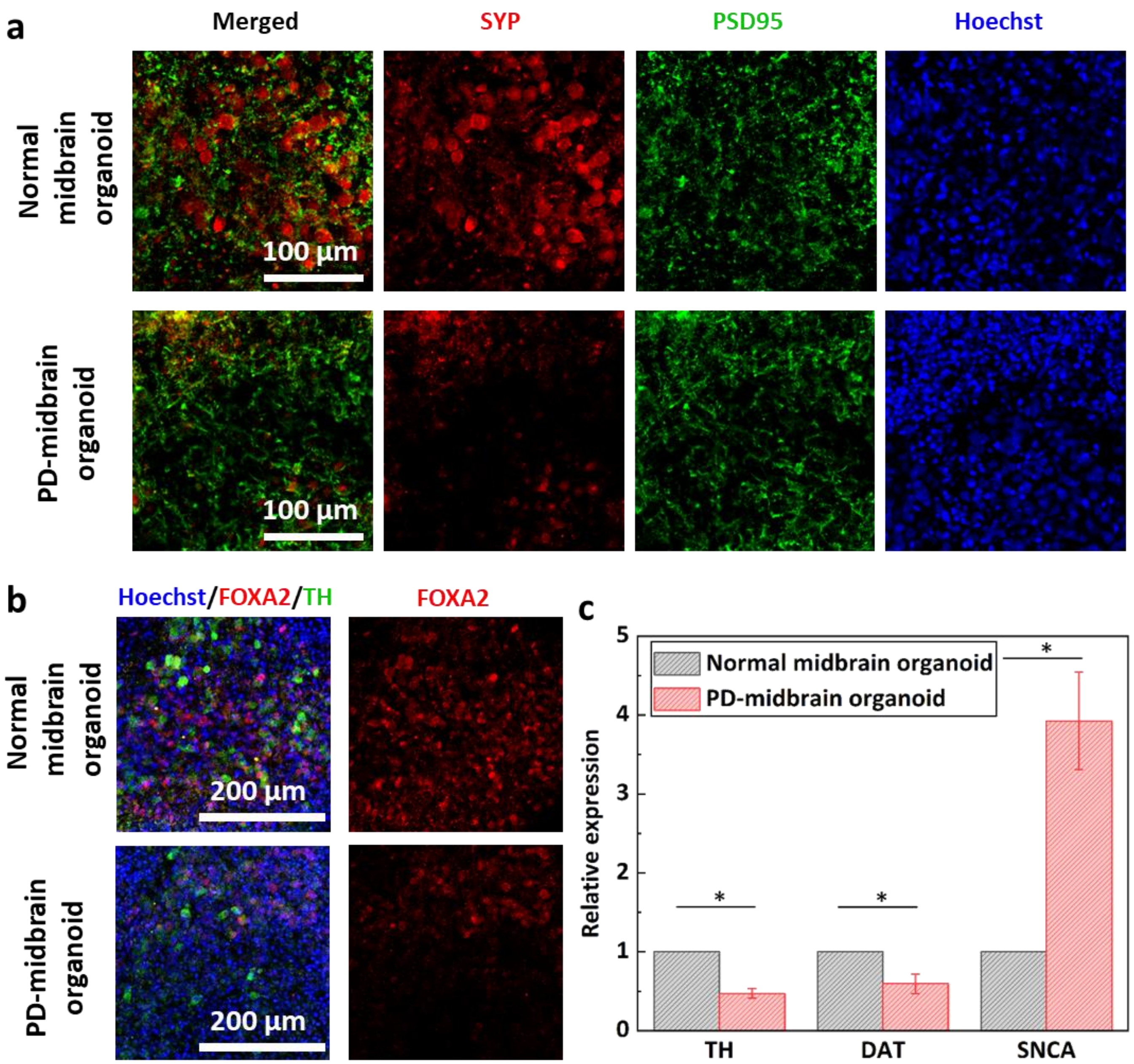

Figure S10. Confirmation of PD-midbrain organoid. a,b) Immunostaining images of normal midbrain and PD-midbrain organoids. c) Normalized expression levels of TH, DAT, and SNCA in normal midbrain and PD-midbrain organoids. *P < 0.05, **P < 0.01, and ***P < 0.001. Error bars correspond to the SD from four measurements.

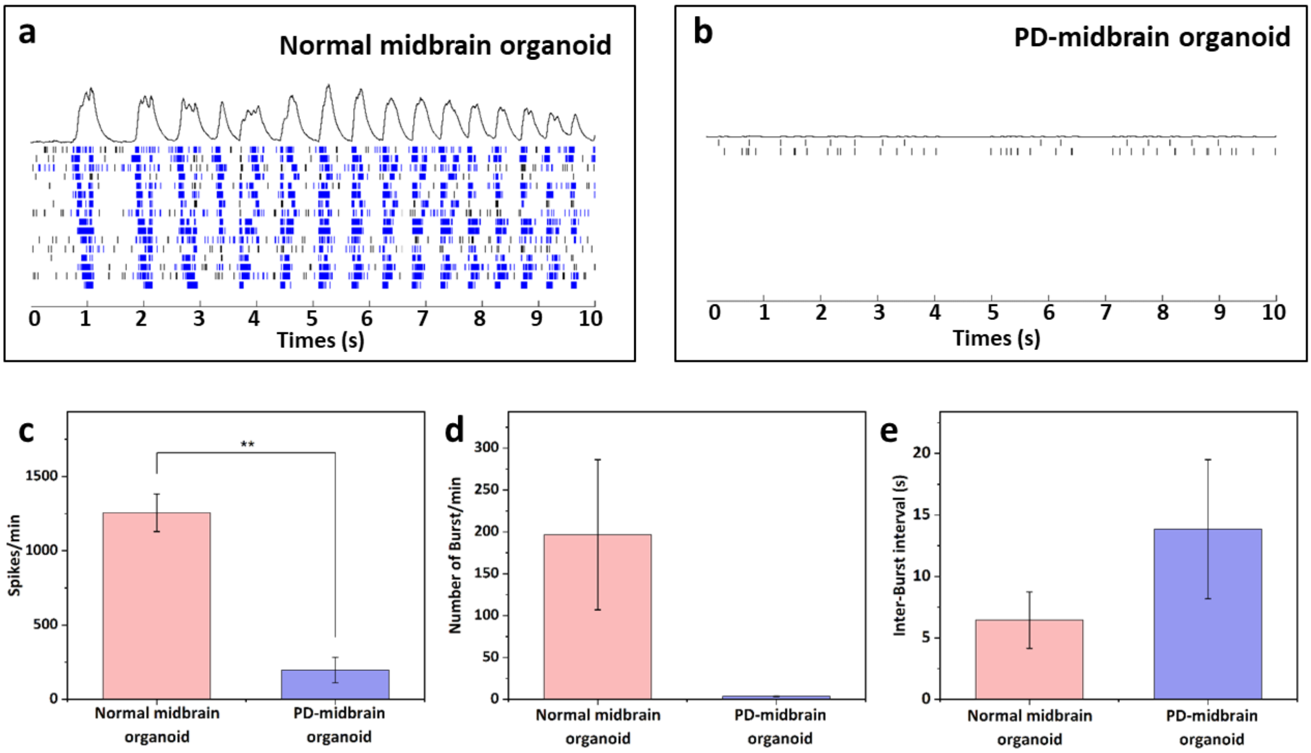

Figure S11. Electrophysiological signals in the midbrain organoid. Spike raster plot of a) normal midbrain and b) PD-midbrain organoids. Electrophysiological quantification of the number of c) number of spikes and d) number of bursts, and e) inter-burst interval of normal midbrain organoid and PD-midbrain organoid. **P < 0.01. Error bars correspond to the SD from four measurements.

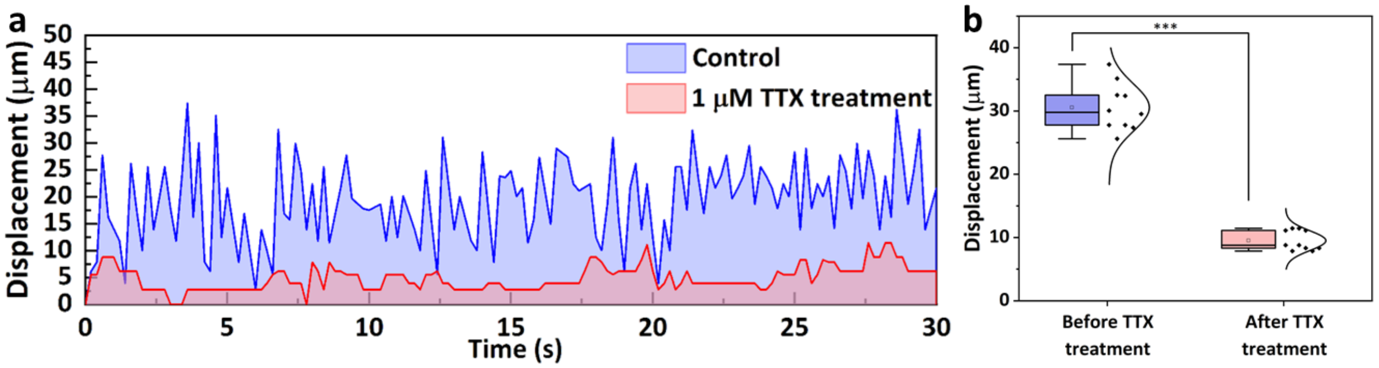

Figure S12. Confirmation of correation between the muscle bundle movement and CNS neural pathway. a) Time-dependent morphological changes in the midbrain organoid. a,b) TTX-induced muscle bundle movement in the cerebral organoid-based biohybrid robot-on-a-chip. ***$P < 0.001$. Error bars correspond to the SD from four measurements.

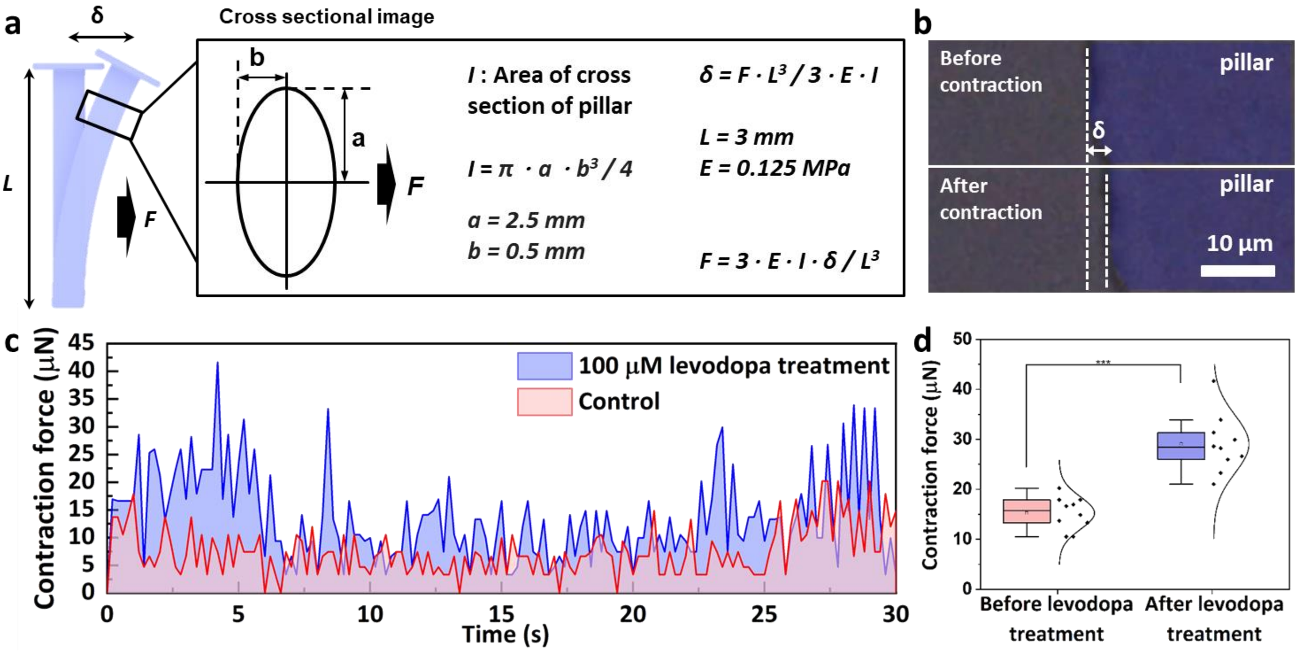

Figure S13. Drug evaluation based on the contraction force of muscle bundle. a) Schematic images of the measurement of muscle bundle contraction using the edge of pillar. b) Optical image of displacement of the edge of pillar by the muscle bundle contraction. c,d) Levodopa-induced muscle bundle contraction in the PD-midbrain organoid-based biohybrid robot-on-a-chip. ***P < 0.001. Error bars correspond to the SD from four measurements.

Table S1. List primers for qPCR

Gene	Forward Primers	Reverse Primers
GAPDH	3'-ATTTCCTGGTATGTGGCTGGG-5'	3'-AAGAGTTGTCAGGGCCCTTTTT-5'
OCT4	3'-AGGAGTAGGGTAAAGGAGGGAA-5'	3'-TCAGGGCCTTGCCACATTAG-5'
PAX6	3'-AGCTCCAAATATAGAAGAGGGGG-5'	3'-ATGATCCGGAGCGACTTCC-5'
TuJ1	3'-CTCTCCCCTCAGCTCACAAG-5'	3'-GAGCAGGGGGAACTTACCAAA-5'
ChAT	3'-CCCACTAGCTGGAATCAGGC-5'	3'-GTACTGCCCATCCAGCCTTT-5'
Islet1	3'-TTGCTTAGCCTGTGCAGACA-5'	3'-TCCATCTGGGAGCTGACACT-5'
SMI-32	3'-GCAGTCCGAGGAGTGGTTC-5'	3'-CGCATAGCGTCTGTGTTCA-5'
MyHC1	3'-CCAGATGAGTGGTGTGACCC-5'	3'-ATCCATGCATGAGTTTCCCCTT-5'
MyoG	3'-GACCCTGGTAACATGGCTCAA-5'	3'-AAGGGAGTGCAGATTGTGGG-5'
Myf5	3'-CGTTATTAGCATATCCCACCACAAC-5'	3'-AAGCTCGGAGGGGAAGGAT-5'
NURR1	3'-CTGTGCGCTGCAAAAGGAG-5'	3'-ATCGTAGACCCCAGTCACATAAC-5'
LMX1A	3'-CATACCAAGGGCCACACACT-5'	3'-TGGGGCAAACAAACCCTTCT-5'
DAT	3'-CATGAGTCCCACGGAGCATT-5'	3'-CACCAGGTATGTCCCTAGCC-5'
TH	3'-ACACTGCTACAACTCACACCA-5'	3'-GGCTGGATAATCGGGAGCTT-5'
SNCA	3'-TGCTCCTCGTCCCTATCTCG-5'	3'-TTAACGTGAGGCGCAAAAGC-5'
